# Evaluation of the comprehensive score for financial toxicity measure in a multicenter cohort of adults with advanced liver disease

**DOI:** 10.1186/s41687-026-01104-2

**Published:** 2026-06-15

**Authors:** Chengbo Zeng, Elizabeth S. Aby, Grace Bizup, Enya Zhu, Ron D. Hays, Maria O. Edelen, Nneka N. Ufere, on the behalf of the PACE-LT Consortium

**Affiliations:** 1 Patient Reported Outcomes, Value and Experience (PROVE) Center, Department of Surgery, Brigham and Women’s Hospital, 1620 Tremont Street, Boston, MA 02120 USA; 2 Division of Transplantation, Department of Surgery, University of Minnesota, Minneapolis, MN USA; 3 Liver Center, Gastrointestinal Division, Department of Medicine, Massachusetts General Hospital, Boston, MA USA; 4 School of Medicine, Duke University, Durham, NC USA; 5 Division of General Internal Medicine, Department of Medicine, University of California, Los Angeles, CA USA; 6 RAND Corporation, Santa Monica, CA USA; 7 RAND Corporation, Boston, MA USA

**Keywords:** Cost, Financial toxicity, Financial burden, Liver disease, Patient-reported outcome (PRO)

## Abstract

**Background:**

This study evaluates the psychometric properties of the COmprehensive Score for financial Toxicity (COST) measure in a multicenter cohort of patients with advanced liver disease (AdvLD).

**Methods:**

Between April 12, 2023, and April 30, 2024, a sample of adults undergoing liver transplantation evaluation was recruited from 13 U.S. transplant centers. We evaluated structural validity using factor analysis and internal consistency reliability using Cronbach’s alpha. Construct validity was assessed by examining correlations between the COST measure, health-related unmet social needs, and EQ-5D-5L scores.

**Results:**

Among the 459 participants, more than half were male (59%), White (82%), and not Hispanic or Latino (77%). Approximately 44% were unable to work due to illness or disability, and over 44% had an annual household income below $50,000. The COST measure demonstrated suboptimal structural validity based on both one- and two-factor models. Cronbach’s alpha for the COST measure was 0.84. The total COST score showed mild to moderate correlations with health-related unmet social needs (r: -0.21 to -0.41) and small correlations with the EQ-5D-5 L visual analogue scale (*r* = 0.24) and pattern scores (*r* = 0.34).

**Discussion:**

Caution may be warranted when applying COST to patients with AdvLD, as suboptimal structural and construct validity compromise score interpretation and clinical utility. Future studies should systematically review evidence on its psychometric properties and evaluate content validity through cognitive interviews with patients with AdvLD.

**Supplementary Information:**

The online version contains supplementary material available at 10.1186/s41687-026-01104-2.

## Introduction

Despite growing recognition of the economic burden of advanced liver disease (AdvLD), no existing measure captures the financial burden on patients with AdvLD and their families [1]. The psychometric properties of the COmprehensive Score for financial Toxicity (COST) measure have been examined in patients with stage IV cancer [2]. It has also been administered to patients with serious non-cancer illnesses; however, evidence regarding its performance among these patients is limited. To determine whether the COST measure performs adequately in patients with serious non-cancer illnesses and whether further adaptation is warranted, we evaluated the psychometric performance of COST in a multicenter cohort of patients with AdvLD. Specifically, we evaluated its structural validity, reliability, and construct validity, which are key measurement properties recommended by the Consensus-based Standards for the selection of Health Measurement Instruments (COSMIN) [3, 4].

## Methods

### Study settings, population, and recruitment

Between April 12, 2023, and April 30, 2024, a convenience sample of adults (≥ 18 years) undergoing liver transplantation evaluation was recruited from 13 U.S. transplant centers (Supplemental Table S1). Patients were included if they were (1) adults (≥ 18 years of age) with a diagnosis of AdvLD (decompensated cirrhosis or hepatocellular carcinoma [HCC]) and (2) able to complete surveys in English or Spanish. Exclusion criteria included prior non-liver solid organ transplant, extrahepatic advanced malignancy, or an uncontrolled neuropsychiatric condition prohibiting the ability to provide informed consent. This study followed the Strengthening the Reporting of Observational Studies in Epidemiology (STROBE) guidelines for cohort studies [5] Written or verbal informed consent was obtained from all participants, and no remuneration was provided for participation.

### COmprehensive Score for Financial Toxicity (COST) measure

We used the 11-item COST measure to assess financial toxicity (Supplemental Table S2). Patients reported their experience with financial issues over the past 7 days using a five-point categorical response scale (0=“not at all”, 1=“a little bit”, 2=“somewhat”, 3=“quite a bit”, 4=“very much”) for each item. The sum score of these 11 items ranged from 0 to 44, with a higher score indicating a lower level of financial toxicity. Sum score was dichotomized for the comparison of demographic and clinical characteristics, with scores < 26 indicating financial toxicity and scores ≥ 26 indicating no/minimal financial toxicity [6].

### Demographic and clinical characteristics

Self-reported demographics, including age, gender, race, ethnicity, marital status, educational level, employment status, and income level, were collected at enrollment. Clinical characteristics, including etiology of liver disease, type of decompensation, Model for End-Stage Liver Disease-Sodium score, and HCC at enrollment, were extracted from the electronic medical record at the time of enrollment through chart review by the research team.

### Statistical analysis

We described the study cohort and compared the differences in demographic and clinical characteristics between patients with no or minimal financial toxicity and those with financial toxicity.

We assessed the structural validity of the COST measure using confirmatory factor analysis (CFA). We used the comparative fit index (CFI, cutoff values > 0.95), root mean square error of approximation (RMSEA, < 0.08), and/or weighted root mean square residual (WRMR, < 1.00) to assess model fit [3, 4] Among these indices, CFI evaluates the improvement of the specified model relative to a baseline model; RMSEA measures the discrepancy between observed and modeled covariance matrices, representing model misfit per degree of freedom; and WRMR quantifies the weighted difference between observed and model-implied residuals when variables are treated as categorical [7]. Consistent with the original development paper, we first examined the one-factor structure [2]. Due to inadequate initial model fit, residual correlations between items were added based on modification indices provided by the statistical software and supported by substantive clinical rationale. Internal consistency was estimated using Cronbach’s alpha, with a value ≥ 0.70 indicating adequate reliability [3, 4]. We evaluated construct validity by examining the correlation of the COST score with health-related unmet social needs (i.e., inability to pay for mortgage [Yes vs. No/Unknown], unstable housing [Yes vs. No/Unknown], worrying about food [Yes vs. No/Unknown], not enough food [Yes vs. No/Unknown], transportation barriers [Yes vs. No/Unknown], and unable to access care [Yes vs. No/Unknown]) and the EQ-5D-5L (i.e., domain, index, and visual analogue scores) [8]. Correlation coefficients were classified as mild (0–0.39), moderate (0.4–0.59), and strong (≥ 0.6) [9]. Factor analysis was conducted using Mplus version 8.1, and all other analyses were performed in SAS version 9.4.

## Results

### Overview

Among the 459 participants, more than half were male (59%, *n* = 269), White (82%, *n* = 376), and not Hispanic or Latino (77%, *n* = 353) (Table 1). Approximately 44% (*n* = 201) were unable to work due to illness or disability, and over 44% (*n* = 204) had an annual household income below $50,000. There was a significant difference in the COST scores between patients with an annual household income below $50,000 and those with higher annual household incomes (10.4 [SD = 12.0] vs.15.2 [SD = 15.3], *p* < 0.001).

**Table 1:** Demographic and clinical characteristics of the analytic samples and by level of financial toxicity (*N* = 459)

Demographic and clinical characteristics	*N* (%)	No/minimal financial toxicity (*n* = 154 [41.7%])	Financial toxicity (215 [58.3%])	*p*-value
Age (median, IQR)	59 (50–65)	62 (55–67)	55 (43–62)	< 0.001
Gender				0.388
Male	269 (59)	95 (61.7)	123 (57.2)	
Female	190 (41)	59 (38.3)	92 (42.8)	
Race				
White	376 (82)	128 (83.1)	181 (84.2)	0.784
African American or Black	14 (3)	3 (2.0)	8 (3.7)	0.323
Other races	58 (13)	16 (10.4)	17 (7.9)	0.410
Missing	11 (2)	NA	NA	NA
Ethnicity				0.284
Hispanic or Latino	103 (22)	30 (19.5)	52 (24.2)	
Not Hispanic or Latino	353 (77)	124 (80.5)	163 (75.8)	
Missing	3 (1)	NA	NA	
Married or living with someone as if married	285 (62)	107 (69.5)	119 (55.4)	0.006
Bachelor’s degree or higher	133 (29)	89 (57.8)	118 (54.9)	0.613
Employment				
Employed (full time or part time)	125 (27)	50 (42.5)	57 (26.5)	0.214
Caring for home or family (not currently working)	9 (2)	2 (1.3)	4 (1.9)	1.000
Unemployed and looking for work	14 (3)	1 (0.7)	7 (3.3)	0.146
Unable to work due to illness or disability	201 (44)	39 (25.3)	118 (54.9)	< 0.001
Retired	105 (23)	57 (37.0)	27 (12.6)	< 0.001
Student	4 (1)	0 (0)	4 (1.9)	0.144
Income level				< 0.001
Less than $25,000	110 (24)	14 (9.1)	21 (9.8)	
$25,000–49,999	94 (20)	16 (10.4)	72 (33.5)	
$50,000–99,999	101 (22)	23 (14.9)	49 (22.8)	
$100,000–149,999	60 (13)	42 (27.3)	44 (20.5)	
Greater than $150,000	45 (10)	59 (38.3)	29 (13.5)	
Missing	49 (11)	NA	NA	
Liver disease etiology				
EtOH	228 (50)	65 (42.2)	119 (55.4)	0.013
MASLD	147 (32)	58 (37.7)	56 (26.1)	0.017
HBV or HCV	59 (13)			
AIH	14 (3)	1 (0.7)	11 (5.1)	0.017
PBC	10 (2)	3 (2.0)	6 (2.8)	0.605
PSC	14 (3)	7 (4.6)	6 (2.8)	0.367
Others	42 (9)	15 (9.7)	23 (10.7)	0.765
Types of decompensation				
Ascites	344 (75)	114 (74.0)	173 (80.5)	0.142
Hepatic encephalopathy	284 (62)	85 (55.2)	148 (68.8)	
EVB	120 (26)	45 (29.2)	57 (26.5)	
Any	392 (85)	130 (84.4)	194 (90.2)	
HCC at Enrollment				0.064
No	358 (78)	117 (76.0)	180 (83.7)	
Yes	98 (21)	37 (24.0)	35 (16.3)	
Missing	3 (1)	NA	NA	
MELD-Na score (Median, IQR)	16 (11–21)	16 (11–20)	16 (12–22)	0.279

Note: ‖: 90 patients without a COST score were excluded. EtOH: ethanol; NASH: non-alcoholic steatohepatitis; HBV: hepatitis B virus; HCV: hepatitis C virus; AIH: autoimmune hepatitis; PBC: primary biliary cholangitis; PSC: primary sclerosing cholangitis; EVB: esophageal variceal bleeding; MELD-Na: model for end-stage liver disease-sodium; HCC: hepatocellular carcinoma

The comparison of demographic and clinical characteristics between patients with and without financial toxicity revealed significant differences in age, marital status, employment status, income level, and liver disease etiology. Patients experiencing financial toxicity were younger (median age: 55 [IQR: 43–62] vs. 62 [55–67] years), more likely to be unable to work due to illness or disability (55% vs. 25%), have an annual income below $50,000 (66% vs. 34%), and/or have alcohol-related liver disease (EtOH; 55% vs. 42%), compared with those reporting no or minimal financial toxicity. In contrast, patients with no or minimal financial toxicity were more likely to be married or living with a partner (70% vs. 55%), retired (37% vs. 13%), and have MASLD as the underlying cause of liver disease (38% vs. 26%), compared with those experiencing financial toxicity.

### Item missing data, structural validity, internal consistency reliability, and construct validity

Nonresponse rates of the 11 items ranged from 6% for item 1 (“I know that I have enough money in savings, retirement, or assets to cover the costs of my treatment”) to 14% for item 9 (“I am concerned about keeping my job and income, including work at home”) (Supplemental Table S2).

**Fig. 1:**
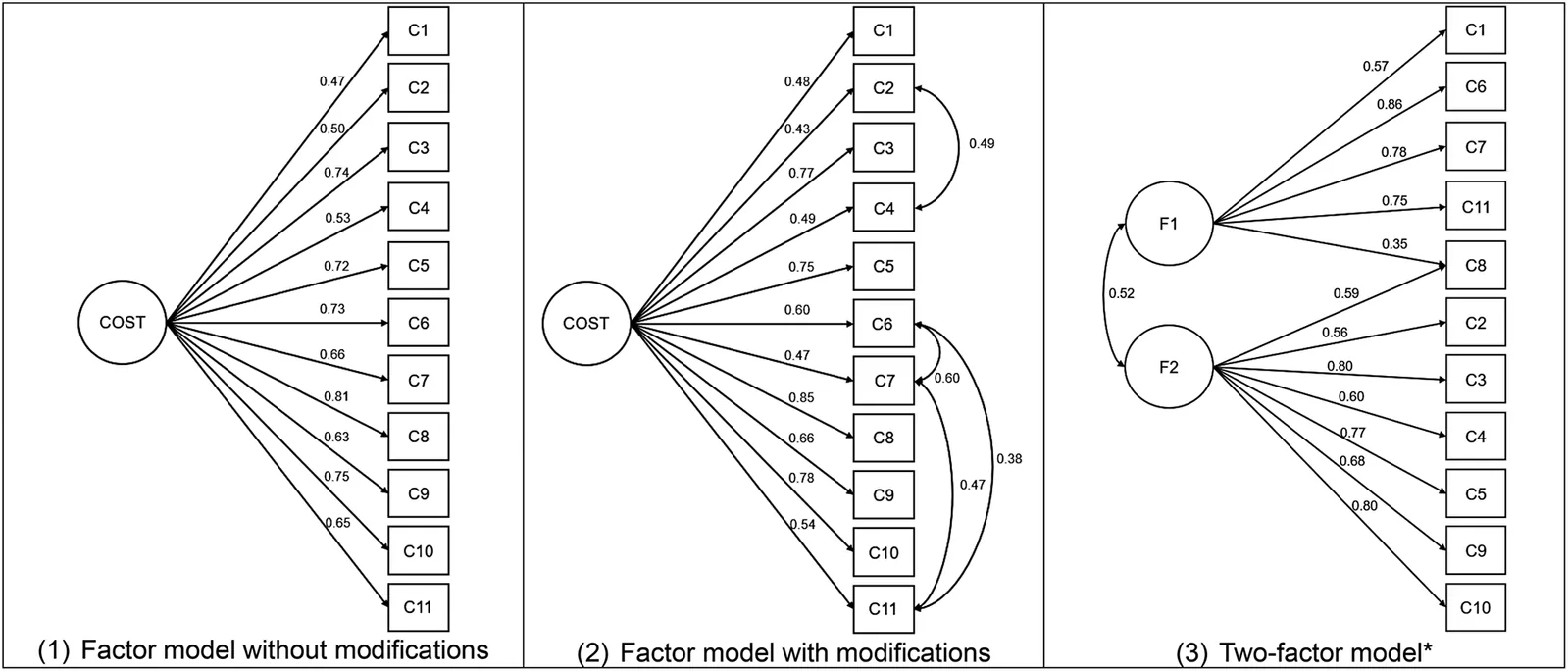
Confirmatory factor analysis of the COST measure among patients with advanced liver disease. Note: COST: Comprehensive Score for Financial Toxicity. To improve model fit, residual correlations between items were added based on modification indices from Mplus version 8.1 and supported by substantive clinical rationale. *: The two-factor model was derived from the exploratory factor analysis

The initial one-factor model demonstrated inadequate fit (overall sample: CFI = 0.86, RMSEA = 0.23, WRMR = 2.42 [Fig. 1]; patients without HCC: CFI = 0.87, RMSEA = 0.23, WRMR = 2.11 [Fig. S1a]; patients with HCC: CFI = 0.82, RMSEA = 0.24, WRMR = 1.28 [Fig. S1b]). The factor loadings ranged from 0.47 for item 1 to 0.81 for item 8 (“I am financially stressed”). After adding residual correlations between item 2 (“My out-of-pocket medical expenses are more than I thought they would be”) and item 4 (“I feel I have no choice about the amount of money I spend on care”), as well as among item 6 (“I am satisfied with my current financial situation”), item 7 (“I am able to meet my monthly expenses”), and item 11 (“I feel in control of my financial situation”), model fit improved but remained suboptimal (overall sample: CFI = 0.93, RMSEA = 0.17, WRMR = 1.66 [Fig. 1]; patients without HCC: CFI = 0.94, RMSEA = 0.17, WRMR = 1.47 [Fig. S1a]; patients with HCC: CFI = 0.91, RMSEA = 0.18, WRMR = 0.94 [Fig. S1b]).

We then evaluated the dimensionality using exploratory factor analysis, which suggested a two-factor solution (Supplemental Tables S3 and S4). Factor 1 (F1) included items 1, 6, 7, and 11, while items 2, 3, 4, 5, 9, and 10 belonged to Factor 2 (F2). Item 8 loaded onto two factors. The subsequent CFA showed a better fit than the other models, but the fit was still suboptimal (overall sample: CFI = 0.95, RMSEA = 0.13, WRMR = 1.40 [Fig. 1]; patients without HCC: CFI = 0.96, RMSEA = 0.13, WRMR = 1.23 [Fig. S1a]; patients with HCC: CFI = 0.93, RMSEA = 0.15, WRMR = 0.88 [Fig. S1b]).

Cronbach’s alpha of the COST measure was 0.84, suggesting acceptable internal consistency reliability. Cronbach’s alphas were similar between patients with HCC (0.82) and those without HCC (0.85).

The total COST score showed statistically significant but weak correlations with the health-related unmet social needs, with correlation coefficients ranging from − 0.21 to -0.41. These associations were generally stronger among patients without HCC compared with those with HCC.

We also found that the total COST score was significantly but weakly correlated with EQ-5D-5 L scores (visual analogue scale [VAS]: *r* = 0.24; index score: *r* = 0.34; domain scores: *r* ranging from − 0.14 to -0.35; Table 2). Stronger correlations between total COST score and EQ-5D-5L scores were found among patients with HCC compared with those without HCC, including the VAS (0.41 vs. 0.18), index score (0.41 vs. 0.30), usual activities (-0.40 vs. -0.31), and pain (-0.42 vs. -0.27). In contrast, correlations between the total COST score and the self-care and anxiety/depression domains were stronger among patients without HCC than among those with HCC.

**Table 2:** Correlations between the COST and EQ-5D-5 L scores for the study cohort and stratified by HCC status

	Overall (*N* = 369)	Patients with HCC (*n* = 72)	Patients without HCC (*n* = 296)
**Health-related unmet social needs**			
Inability to pay for mortgage	-0.37***	-0.30*	-0.37***
Unstable housing	-0.34***	-0.33**	-0.35***
Worrying about food	-0.41***	-0.24*	-0.44***
Not enough food	-0.38***	-0.37**	-0.38***
Transportation barriers	-0.33***	-0.31**	-0.34***
Unable to access care	-0.21***	-0.17	-0.21***
**EQ-5D-5 L scores**			
Visual analogue scale	0.24***	0.41***	0.18**
Patten score	0.34***	0.41***	0.30***
Mobility domain	-0.14**	-0.20	-0.10
Self-care domain	-0.19***	-0.04	-0.20***
Usual activities domain	-0.35***	-0.40***	-0.31***
Pain domain	-0.31***	-0.42***	-0.27***
Depression/anxiety domain	-0.35***	-0.29*	-0.35***

Note: ‖: Only patients with a COST score were included in the correlation analysis. One patient without HCC enrollment was excluded. HCC: hepatocellular carcinoma. *: *p* < 0.05; **: *p* < 0.01; ***: *p* < 0.001

## Discussion

The COST measure was developed through a rigorous, iterative process, but was originally designed using a sample of patients with stage IV solid cancers who were receiving chemotherapy [2]. Semi-structured interviews with patients and clinicians ensured it captured the financial burden of cancer and its treatment. Although support for its psychometric properties has been shown in advanced cancer populations, it showed suboptimal structural validity in this study of patients with AdvLD, including those with HCC.

Content and structural validity are crucial when applying existing measures to new contexts, particularly those developed for a specific disease but intended for broader use [3, 4]. In our analyses, replication of the factor structure reported in the original development study resulted in an acceptable CFI but substantial residual error, even after allowing residual correlations between selected items. This finding suggests that the underlying dimensional structure of the COST measure may differ when applied to those with severe non-cancer illnesses, potentially due to differences in disease progression and treatment contexts [2, 10]. Consistent with this possibility, a prior study in patients with diabetes reported that a two-factor model provided a better fit and demonstrated acceptable psychometric performance [10]. In our study, the two-factor model, which reflected the general financial situation (items 1, 6, 7, and 11) and the impact of AdvLD on financial situation (items 2, 3, 4, 5, 9, and 10), still had substantial residual error, and item 8 loaded onto two factors. This suggests that some items need to be adapted to the context of non-cancer severe illnesses and associated disease progression and treatment.

The COST score showed suboptimal construct validity. We found only mild to moderate correlations between the COST score and health-related unmet social needs. Additionally, while previous studies found that the COST score was strongly correlated with quality of life in cancer populations [11, 12] our secondary analyses found only a small correlation. Together, these findings suggest that the COST measure may capture aspects of financial burden that relate differently to health-related unmet social needs and quality of life in patients with AdvLD and other non-cancer severe illnesses, highlighting the need for further evaluation of its measurement properties in these populations.

There are some limitations that need to be acknowledged. First, although this study cohort was recruited from 13 U.S. transplant centers, it may be biased by the nature of convenience sampling. Second, our CFA was based on multiple post-hoc modifications and/or exploratory factor analysis; it might suffer from overfitting. Therefore, caution is needed when interpreting the findings from our CFA.

In conclusion, caution is needed when applying COST to patients with AdvLD, as suboptimal structural and construct validity may compromise score interpretation and clinical utility. Future studies should systematically review the quality of evidence on its psychometric properties and evaluate content validity through cognitive interviews with patients with AdvLD.

## Supplementary Information

Below is the link to the electronic supplementary material.

Supplementary Material 1

## Data Availability

Individual patient data are not available for sharing.

## Abbreviations

AdvLD: Advanced liver disease
CFA: Confirmatory factor analysis
CFI: Comparative fit index
COSMIN: Consensus-based Standards for the selection of Health Measurement Instruments
COST: COmprehensive Score for financial Toxicity
HCC: Hepatocellular carcinoma
RMSEA: Root mean square error of approximation
SD: Standard deviation
VAS: Visual analog scale
WRMR: Weighted root mean square residual
