# Supplementary material for: Evaluation of the comprehensive score for financial toxicity measure in a multicenter cohort of adults with advanced liver disease
Source: J Patient Rep Outcomes. 2026 Jun 15;10:153. doi: 10.1186/s41687-026-01104-2 (PMC13500444; doi:10.1186/s41687-026-01104-2)
Supplement: Supplementary file 1 [file 41687_2026_1104_MOESM1_ESM.docx]

**Evaluation of the Comprehensive Score for Financial Toxicity Measure in a Multicenter Cohort of Adults with Advanced Liver Disease**

**Supplement Table S1.** Study site, region, and number of participants

| **Site** | **Region** | **Number of participants (N [%])** |
| --- | --- | --- |
| S1 | Northeast | 100 (21.8) |
| S2 | Northeast | 25 (5.4) |
| S3 | Northeast | 24 (5.2) |
| S4 | Northeast | 28 (6.1) |
| S5 | Midwest | 50 (10.9) |
| S6 | Midwest | 20 (4.4) |
| S7 | Southeast | 12 (2.6) |
| S8 | Southeast | 20 (4.4) |
| S9 | Southeast | 24 (5.2) |
| S10 | Southwest | 29 (6.3) |
| S11 | Midwest | 34 (7.4) |
| S12 | West | 50 (10.9) |
| S13 | West | 43 (9.4) |

**Table S2.** COST Items and patterns of item response in patients with advanced liver disease

| Below is a list of statements that other people with your illness have said are important. **Please circle or mark one number per line to indicate your response as it applies to the past 7 days.** | | | | | | |
| --- | --- | --- | --- | --- | --- | --- |
| Items | Missing | Not at all | A little bit | Somewhat | Quite a bit | Very much |
| 1.I know that I have enough money in savings, retirement, or assets to cover the costs of my treatment | 27 (5.9) | 151 (32.9) | 78 (17) | 81 (17.7) | 52 (11.3) | 70 (15.3) |
| 2.My out-of-pocket medical expenses are more than I thought they would be* | 28 (6.1) | 41 (8.9) | 40 (8.7) | 82 (17.9) | 67 (14.6) | 201 (43.8) |
| 3.I worry about the financial problems I will have in the future as a result of my illness or treatment* | 28 (6.1) | 98 (21.4) | 83 (18.1) | 80 (17.4) | 73 (15.9) | 97 (21.1) |
| 4.I feel I have no choice about the amount of money I spend on care* | 32 (7) | 84 (18.3) | 81 (17.7) | 90 (19.6) | 65 (14.2) | 107 (23.3) |
| 5.I am frustrated that I cannot work or contribute as much as I usually do* | 28 (6.1) | 136 (29.6) | 77 (16.8) | 66 (14.4) | 45 (9.8) | 107 (23.3) |
| 6.I am satisfied with my current financial situation | 28 (6.1) | 147 (32) | 73 (15.9) | 105 (22.9) | 47 (10.2) | 59 (12.9) |
| 7.I am able to meet my monthly expenses | 32 (7) | 50 (10.9) | 58 (12.6) | 89 (19.4) | 83 (18.1) | 147 (32) |
| 8.I am financially stressed* | 29 (6.3) | 69 (15) | 54 (11.8) | 99 (21.6) | 80 (17.4) | 128 (27.9) |
| 9.I am concerned about keeping my job and income, including work at home* | 65 (14.2) | 42 (9.2) | 57 (12.4) | 47 (10.2) | 189 (41.2) | 65 (14.2) |
| 10.My liver disease**^‡^** or treatment has reduced my satisfaction with my present financial situation* | 34 (7.4) | 78 (17) | 67 (14.6) | 77 (16.8) | 59 (12.9) | 144 (31.4) |
| 11.I feel in control of my financial situation | 31 (6.8) | 112 (24.4) | 86 (18.7) | 95 (20.7) | 53 (11.6) | 82 (17.9) |

*: Recoded items

‡: This item was modified from the original by replacing “cancer” with “liver disease.”

**Table S3.** Eigenvalues for the simple correlation matrix in exploratory factor analysis

|  | Eigenvalues |
| --- | --- |
| 1 | 5.10 |
| 2 | 1.88 |
| 3 | 0.87 |
| 4 | 0.65 |
| 5 | 0.51 |
| 6 | 0.44 |
| 7 | 0.42 |
| 8 | 0.37 |
| 9 | 0.31 |
| 10 | 0.25 |
| 11 | 0.21 |

**Table S4.** Promax rotated factor loadings in the exploratory factor analysis

| Items | **Factor 1** | **Factor 2** |
| --- | --- | --- |
| 1.I know that I have enough money in savings, retirement, or assets to cover the costs of my treatment | 0.57 | -0.04 |
| 2.My out-of-pocket medical expenses are more than I thought they would be* | -0.17 | 0.69 |
| 3.I worry about the financial problems I will have in the future as a result of my illness or treatment* | 0.15 | 0.74 |
| 4.I feel I have no choice about the amount of money I spend on care* | -0.28 | 0.84 |
| 5.I am frustrated that I cannot work or contribute as much as I usually do* | 0.26 | 0.61 |
| 6.I am satisfied with my current financial situation | 0.82 | 0.06 |
| 7.I am able to meet my monthly expenses | 0.83 | -0.12 |
| 8.I am financially stressed* | 0.49 | 0.55 |
| 9.I am concerned about keeping my job and income, including work at home* | 0.18 | 0.59 |
| 10.My liver disease or treatment has reduced my satisfaction with my present financial situation* | 0.26 | 0.63 |
| 11.I feel in control of my financial situation | 0.70 | 0.11 |

*: Recoded items

|   **S1a.** Factor model without modifications |   **S1a.** Factor model with modifications |   **S1b.** Two-factor model* |
| --- | --- | --- |

|   **S1b.** Factor model without modifications |   **S1b.** Factor model with modifications |   **S1b.** Two-factor model* |
| --- | --- | --- |

**Figure S1.** Confirmatory factor analysis of the COST measure among patients with advanced liver diseases stratified by HCC status

**Note:** HCC: hepatocellular carcinoma**.** Figure S1a provides results for patients without HCC while Figure S1b provides results for patients with HCC. COST: Comprehensive Score for Financial Toxicity. To improve model fit, residual correlations between items were added based on modification indices from Mplus version 8.1 and supported by substantive clinical rationale. *: The two-factor model was derived from the exploratory factor analysis.
